# Manifolds for the neural representation of 3D spatial experience

**DOI:** 10.64898/2026.10.22.760836

**Published:** 2026-10-27

**Authors:** Raymond Doudlah, Mengyu Xiao, Satchal Postlewaite, Byounghoon Kim, Noah J. Cowan, Ari Rosenberg

## Abstract

The precision with which animals navigate and interact with objects depends in part on how their brains represent position and orientation in three dimensions. A central challenge is to explain within a common framework how neural systems ranging from insects to primates represent three-dimensional (3D) pose. Here we develop a framework based on the hypothesis that neural representations of pose lie on manifolds derived from the Special Euclidean Group, SE(3), which describes the full space of rigid-body positions and orientations in three dimensions. The framework shows how movement constraints, object geometry, and visual features jointly determine the topology of the resulting pose manifold, yielding a taxonomy that guides experimental tests. We apply the framework to derive low-dimensional manifolds for neural representations of 3D object orientation in macaque parietal cortex and head direction in the limbic systems of fruit bats, mice, and monkeys. This analysis also guides the construction of neuronal tuning functions that respect the topology determined by experimental conditions. Together, these results provide a common approach for linking the geometry of 3D pose to neural coding across species and brain systems.

**Significance statement:**

Behaviors such as reaching and grasping are guided by visual estimates of 3D object pose, whereas postural control and navigation depend on multisensory estimates of how the self is posed in the world. Despite being resolved by distinct neural circuits, we propose that these computational goals share a common topology which we use to develop a unified mathematical framework for describing 3D spatial experience. The framework accounts for neuronal representations found across distinct sensorimotor and cognitive systems, and multiple species. Through the application of geometric topology, this work reveals formal equivalencies across disparate neural systems and research domains, and outlines a deductive strategy for testing theories of neuronal coding.

**Declaration of competing interests:**

The authors declare no competing interests.

